# Self-Enhanced Mixed Attention Network for Three-Modal Images Few-Shot Semantic Segmentation

**DOI:** 10.3390/s23146612

**Published:** 2023-07-22

**Authors:** Kechen Song, Yiming Zhang, Yanqi Bao, Ying Zhao, Yunhui Yan

**Affiliations:** 1 School of Mechanical Engineering & Automation, Northeastern University, Shenyang 110819, China; songkc@me.neu.edu.cn (K.S.); zhangyiming@stumail.neu.edu.cn (Y.Z.); zhaoying@stumail.neu.edu.cn (Y.Z.); yanyh@mail.neu.edu.cn (Y.Y.); 2 National Key Laboratory for Novel Software Technology, Department of Computer Science and Technology, Nanjing University, Nanjing 210023, China

**Keywords:** multi-modal images, few-shot semantic segmentation, three-modal registration

## Abstract

As an important computer vision technique, image segmentation has been widely used in various tasks. However, in some extreme cases, the insufficient illumination would result in a great impact on the performance of the model. So more and more fully supervised methods use multi-modal images as their input. The dense annotated large datasets are difficult to obtain, but the few-shot methods still can have satisfactory results with few pixel-annotated samples. Therefore, we propose the Visible-Depth-Thermal (three-modal) images few-shot semantic segmentation method. It utilizes the homogeneous information of three-modal images and the complementary information of different modal images, which can improve the performance of few-shot segmentation tasks. We constructed a novel indoor dataset VDT-2048-5^i^ for the three-modal images few-shot semantic segmentation task. We also proposed a Self-Enhanced Mixed Attention Network (SEMANet), which consists of a Self-Enhanced module (SE) and a Mixed Attention module (MA). The SE module amplifies the difference between the different kinds of features and strengthens the weak connection for the foreground features. The MA module fuses the three-modal feature to obtain a better feature. Compared with the most advanced methods before, our model improves mIoU by 3.8% and 3.3% in 1-shot and 5-shot settings, respectively, which achieves state-of-the-art performance. In the future, we will solve failure cases by obtaining more discriminative and robust feature representations, and explore achieving high performance with fewer parameters and computational costs.

## 1. Introduction

In recent years, many computer vision tasks have promoted dramatic advances, including salient object detection [1,2,3,4,5], object detection [6,7,8], and semantic segmentation [9,10,11]. Among them, semantic segmentation is gradually becoming a research hotspot. Based on the deep convolutional neural networks, many methods such as dilated convolutions [12] were proposed. With the iterative development of these methods, the semantic segmentation performance is further improved.

Fully supervised semantic segmentation works [13,14,15,16,17] based on deep learning have achieved satisfactory results, even if they only use single-modal image as their model’s input. Despite the structure of deep networks being effective, it still has some limitations that the model needs a large number of annotated examples. Especially for pixel-level prediction tasks like semantic segmentation, the cost of data is heavy, and it requires substantial human effort. To reduce the dependence on data, many methods have been proposed that do not require all images to be labeled [18,19,20,21]. These approaches alleviate the data-hunger issue effectively, but the models do not have enough generalization capability with the limited annotated training images. The few-shot semantic segmentation methods still can develop the generalization capability with limited examples and achieve state-of-the-art results [22,23].

However, under insufficient illumination or complex working conditions, the visible image cannot provide enough information. Without enough information, the performance of the model to be not satisfactory as we expected. To deal with this challenge, many works proposed using Visible-Depth/Visible-Thermal (two-modal) images as their model’s input [24,25,26,27,28]. The input of other modality provides complementary information to the visible images (V) so that the model can tackle the more difficult scenarios. To accommodate the limited number of two-modal images, the two-modal few-shot methods [11,29,30] are proposed. They not only propose the method, but also produce the corresponding dataset, respectively. Unfortunately, their datasets also have some flaws. Usually, the dataset should contain 20 categories. But the dataset of [31] only has 9 categories, and [12] set up the dataset with 16 categories. There are still some problems with the previous work. Depth images (D) can provide depth information, but fail when objects stay close together. Thermal infrared images (T) can provide greater support in nighttime scenes, but struggle to provide valuable information when objects are at a similar temperature.

To capture as much information as possible in the same scene, we construct our dataset VDT-2048-5^i^. The original dataset VDT-2048 [32] has 2048 sets of three-modal images. We select 1037 sets images of original dataset and divide it into 20 categories as usual settings.

In the process of shooting the dataset, we find that there is no three-modal camera available directly, so we build a shooting platform ourselves using two cameras. Because the two cameras do not have the same field of view, we perform registration work on them. Before that, there is a lot of good work on two-modal calibration [33,34] and camera registration [35,36]. We also gained a lot of help from these methods.

For the problem setting, we keep in line with the existing methods. Support images are images with annotations, while query images are images without annotations. We present our model with 1-shot scenario, each query image from the novel class has only one support image (images with annotations).

**Figure 1:**
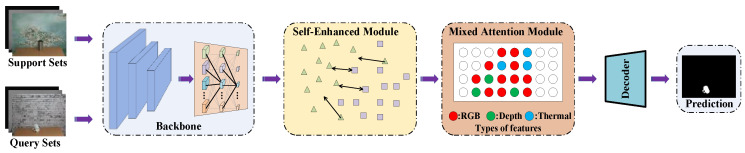
This is a brief diagram of the overall structure of SEMANet proposed by us.

We propose a novel Self-Enhanced Mixed Attention Network (SEMANet), which consists of a backbone network, a self-enhanced module, and a mixed attention module as shown in Figure 1. We use the same backbone network as HSNet [37], the network first extract features from different convolution layers, then exploits diverse semantic/geometric information representations through 4D correlation calculation. We use multi-scale convolution layers to process high-level semantic information and low-level geometric information, finally output predictions in a top–down manner.

The self-enhanced module amplifies the difference between the foreground feature and the background feature of the single modality and strengthens the weak connection for the foreground features at the same time. Then, the feature of three single modality is concatenated and fed into the mixed attention module. In SE module, the foreground feature is further enhanced and the noises of the feature are filtered. The SE module also obtains the core information and overlay the information on the original feature, which can strengthen the three-modal feature. When the features are processed from the mixed attention module, a better feature representation is obtained based on the complementary information between the three modalities. The decoder of the system uses the better feature obtained by the MA module to obtain the prediction.

Extensive comparative experiments are conducted on the novel three-modal dataset, VDT-2048-5^i^, which can verify the validity of our model. The dataset contains many images under insufficient illumination, and most of the foreground objects do not account for a large percentage, which is a great challenge to the model.

The main contributions of this paper are summarized as follows:

(1) As far as we know, it is the first time that three-modal images are used as input for few-shot semantic segmentation. The effectiveness of the three-modal fusion mechanism designed by us is proved by ablation experiments.
(2) We make a novel V-D-T few-shot semantic segmentation dataset VDT-2048-5^i^ and conduct many comparison experiments with the existing methods. We also prove that the effectiveness and necessity of three-modal few-shot semantic segmentation.
(3) We design a novel Self-Enhanced Mixed Attention Network (SEMANet). It includes an SE (Self-Enhanced) module for enhancing three-modal features and a MA (Mixed Attention) module for fusing three-modal features. The experimental results prove that the proposed SEMANet achieves the state-of-the-art performance.

The rest of this article is organized as follows. In Section 2, we review the related work of natural image segmentation and small sample semantic segmentation based on CNN. In Section 3, we elaborate on our method. In Section 4, we describe the construction process of data set. In Section 5, we present experiments, ablation studies, and visual analysis. In Section 6, we summarize this conclusion.

## 2. Related Work

### 2.1. Single-Modal Segmentation

Semantic Segmentation is an important task in the field of computer vision, which aims to assign each pixel in an image to a predefined semantic category, so as to realize pixel-level understanding and segmentation of an image. In recent years, semantic segmentation methods have made great progress based on deep learning technology [38]. Rehman et al. [39] proposed an encoder–decoder-based model named BrainSeg-Net to solve the problem of deep information loss. The 3D U-Net [40] provides faster, higher accuracy, and more consistent segmentation across GAs. RAAGR2-Net [41] aims at minimizing the loss of information during depth feature extraction. A novel DE-ResUnet based on texture features and background knowledge is proposed by Wu et al. [42] for brain tissue segmentation. Because thermal infrared information [43] is not affected by illumination changes and extreme weather, semantic segmentation using thermal images has attracted great attention. Maheswari et al. [44] presented a top–down attention and gradient alignment-based graph neural network (AGAGNN) to discover the crucial semantic information. EdgeFormer was proposed by Wang et al. [45] to promote the segmentation performance in electrical equipment’s edges and interiors. CEKD [46] transfers the edge detection capability of an RGB-T teacher network to a thermal-only student network.

### 2.2. Multi-Modal Segmentation

The introduction of multi-modal images into semantic segmentation has gradually become the main means. Yadav et al. [47] segmented the RGB-D images using Random Henry gas solubility optimization-fuzzy clustering (RHGSO-FC). Zhang et al. [48] proposed a multi-scale network for multi-level feature fusion of two-stream inputs to perform the final segmentation. At the same time, high-level features are used to output the corresponding loss function to help the model identify the differences between different modes, which achieved great results in the same dataset as MFNet [49]. PST900 [34] proposed a dataset based on underground scenarios, and proposed a multi-stream input network to add additional sources of information to the model, achieving good results. HeatNet [50] proposed a teacher–student network to reduce parameters, and introduced a dataset based on street view. Lan et al. [51] proposed a two-stage network, which first extracts single-mode features, and then merges and improves them in the second stage. Experiments on two RGB-T image segmentation datasets [49,51] show that this method can effectively extract cross-pattern features to complete interaction, and significantly reduce the number of model parameters.

For the methods which use depth images as auxiliary information, Wang et al. [52] introduce a deconvolutional neural network to model specific features, which greatly improved the segmentation accuracy of the model. Jiang et al. [53] propose an encoder–decoder network structure that uses a residual block to avoid the model degradation problem. They also carry out separate supervised learning of different layers of the decoder to better optimize the parameters of the model. They achieve good results on indoor RGB-D semantic segmentation. Wu et al. [54] propose a network that can integrate the geometric constraint into the conventional receptive field. With a depth-aware contextualized attention module, the network can improve the convolution and not introduce extra learning parameters. It obtains satisfactory results on both indoor and outdoor RGB-D semantic segmentation benchmarks.

### 2.3. Few-Shot Semantic Segmentation

In recent years, few-shot learning has been used to solve the problem of the limited samples in semantic segmentation, and has significantly improved the performance of the model. OSLSM [31] proposed the rule of few-shot semantic segmentation and introduces a double-branch structure to transfer the knowledge of the support sets to the query sets. CANet [55] proposed a double-branch network, which uses the shared weights backbone to extract features, and finally uses iterative modules to improve the segmentation results step by step. PGNet [56] proposed a graph network based on the attention mechanism, which establish an effective long-range context relationship. The model can more effectively extract the knowledge from the support set to guide the model. PFENet [57] leverages high-level features and introduces the prior acknowledgment to improve the accuracy of the model and retain high generalization. ASGNet [58] uses the superpixel-guided mechanism to cluster the prototype feature and use the prototype to guide the model to allocation. HSNet [37] proposed a center-pivot 4D Conv kernel and stacked the kernels to deal with the correlation of different scales. It achieves real-time inference. ASNet [59] proposed the attentive squeeze network which uses stridden global self-attention to squeeze semantic correlations into a foreground map. It creates state-of-the-art performance.

In addition, Zhang et al. [29] introduce depth images into the model to provide additional information, and uses late fusion to improve the segmentation accuracy. V-TFSS [11] proposed a network for visible and thermal images. The network analyzes the difference between visible and thermal images, and proposes different prototypes to guide segmentation. These works have a great inspiration for our work.

## 3. Methods

### 3.1. Problem Setting

According to the rules set by Shaban et al. [31], few-shot semantic segmentation task consists of two datasets: the base class dataset $D_{base}$ and the novel class dataset $D_{novel}$. Our model is trained on the base class dataset $D_{base}$, obtains the corresponding knowledge, and tests on the novel class dataset $D_{novel}$ on this basis. All the samples are divided into two parts, $C_{base}$ and $C_{novel}$. $C_{base}$ only appear in the base class dataset $D_{base}$, while $C_{novel}$ only appear in novel class dataset $D_{novel}$, which mean $C_{base}\cap C_{novel}=Ø$. The specific settings of the dataset are as follows:

(1) $D_{base}={\left(x_{i}^{rgb},x_{i}^{th},x_{i}^{d},y{\left(C\right)}_{i}\right)}_{i=1}^{N}$, $x_{i}^{rgb}$ represents the visible image, and $x_{i}^{th}$ represents the infrared image, $x_{i}^{d}$ represents the depth image, and all three are pixel-level corresponding pictures of the same scene. $y{\left(C\right)}_{i}$ represents the corresponding mask of the base class images. $C\left(C\subset C_{base}\right)$ represents the base class in the training, and N represents the number of base images.
(2) $D_{novel}={\left(x_{j}^{rgb},x_{j}^{th},x_{j}^{d},y{\left(C\right)}_{j}\right)}_{j=1}^{n}$, n represents the number of sets of visible, infrared, and depth image pairs. By comparing the predicted results of the model with its corresponding ground truth image, we can test the performance of the model.

To sum up, the goal of few-shot semantic segmentation is to train the segmentation and generalization ability of the model on the base class dataset $D_{base}$, so as to perform the segmentation of novel class dataset $D_{novel}$ that have never been seen before. In the process of training, the model will randomly divide the samples of the same category into support set and query set. According to the corresponding settings, the model assigns a corresponding number of support images to each query image. During the testing phase, the model performs semantic segmentation tasks on novel class that has never been seen before to test the performance of training on the base class.

In general, if K annotated support set images are assigned to each query image, we describe this type of problem as K−shot segmentation task, usually the few-shot methods employ 1−shot and 5−shot. If there are C semantic categories in a single image, then we describe this type of problem as C−way segmentation task. This paper uses 1−way segmentation task.

### 3.2. The Proposed Model

**Figure 2:**
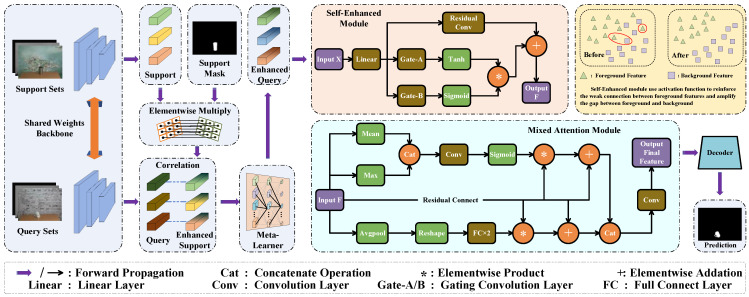
The overall architecture of our SEMANet, which includes the self-enhanced module (SE) and the mixed attention module (MA).

As shown in Figure 2, we propose a novel network for three-modal few-shot semantic segmentation tasks. We use images of three modalities in the same scene to compensate for the lack of information in single-modal scenes and to further improve the performance of two-modal scenes. We present two main modules, the self-enhanced module, and the mixed attention module. The former uses different convolutional layers to enhance the similar feature information of the same modality, it also amplifies the differences between the foreground feature and the background feature. The latter uses a dual attention mechanism to fuse the information among the three modalities, thus enhancing the representativeness of the correspondence between the query samples and the support samples. So, it can provide a better feature representation for the decoder of the network.

### 3.3. Feature Extraction

In the process of extracting the features of the three modalities, we consider that there is no large variability, so we use the same encoder for feature extraction. For the images of support set and query set, we use a decoder with shared weights (because the features of the support images and the query images are highly correlated). Here, we use the pre-trained ResNet-50 [60] to extract the feature. During the process of training, in order to reduce the number of learnable parameters of the model, we freeze the parameter updates of the backbone network.

As mentioned in HSNet [37], we compute the similarity between features by using deeply stacked 4D convolutional layers to gradually aggregate the relevant information from the input into a global context. We gradually compress its dimensionality, and then process the correlation to derive more representative features for subsequent operations. We use the same backbone network to extract the features of the three modalities, then let the features interact with their corresponding mask to activate the foreground region.

We obtain the enhanced support set features, and compute the correlation with the query set features which obtained from the decoder. Because of the large differences between the three modalities, the support image and the query image features are computed between the same modality, and the cross-modal correlation is not involved here.

### 3.4. Self-Enhanced Module

The self-enhanced module is mainly used to obtain the corresponding feature matrix by aggregating and searching the features in different directions. The matrix is used as a parameter to enhance the features of the model. The features of the three modalities will be handled separately at this stage. The following is a brief introduction of the single-modal features as an example.

The corresponding relationships between the support images and the query images are processed by the meta-learner, which we call enhanced features. It will be fed into the convolutional layer through the linear layer. The features will pass through two gating layers. The two convolutional layers will perform an aggregated search in different directions of the features using conv1d and conv2d to obtain the corresponding feature matrices $\alpha_{1}$ and $\alpha_{2}$. We adjust the parameters of the convolutional layer; they can effectively cover all positions of the original features without missing the relevant information at the edges.

(1) $$\alpha_{1}=\left(\theta_{1}x+b\right),$$

(2) $$\alpha_{2}=\left(\theta_{2}x+c\right),$$

After obtaining the corresponding feature matrices, $\alpha_{1}$ and $\alpha_{2}$ are put through different activation functions, Sigmoid activation function is used to strengthen the weak connection among the foreground feature, Tanh activation function is used to amplify the gap between the foreground feature and the background feature. Then, we obtain two parameter matrices, assign different weight coefficients $m_{1}$ and $m_{2}$ to the two matrices, and obtain the corresponding parameter matrices $\beta_{1}$ and $\beta_{2}$.

(3) $$\beta_{1}=m_{1}\times \left(tanh(\alpha_{1})\right),$$

(4) $$\beta_{2}=m_{2}\times \left(Sigmoid(\alpha_{2})\right),$$

After that, the obtained $\beta_{1}$ and $\beta_{2}$ are multiplied at the element level to obtain the final parameter matrix δ. Because the foreground region and the background region are relatively distinct in our dataset, we assign the same weights to two feature matrices in our experiments, $m_{1}$ and $m_{2}$ are set to 1.0.

(5) $$\delta =\beta_{1}\times \beta_{2}$$

Finally, unlike the previous work [61], we abandon the graph convolution layer to avoid the excessive amount of model parameters, it can also avoid the convolution layer that cannot converge better because of the few samples. We add δ with the features convolved with residual convolution, elemental multiplication is not used to avoid excessive damage to the original features, which affects the effect of cross-modal fusion in the next stage.

(6) F=δ+Residual(x),

Residual(·) represents the residual convolution, + represents the summation at the element level. After this series of operations, we amplify the difference between the foreground feature and the background feature of the single modality. We also strengthen the weak connection for the foreground features at the same time, which can prevent the feature from missing in the subsequent fusion process.

After passing the self-enhanced module, we obtain the enhanced feature representations of the three different modalities. We concatenated them in the channel dimension and used them as input for the next stage.

### 3.5. Self-Enhanced Module

The features F obtained from the self-enhanced module will pass through two sub-modules of the mixed attention module, respectively, for the further fusion of features.

In the spatial attention module, the feature F will pass through the mean and the maximum operation, respectively, the former is used to filter out the noises and obtain the overall situation of the features, while the latter enhances the foreground features. We concatenate them and send them to the convolutional layer for further information extraction, Sigmoid activation function is used to sparse the distance between the foreground feature and the background feature, then obtain the weight coefficient $\gamma_{1}$.

(7) $$\gamma_{1}=Sigmoid(Cat(Mean(F),Max(F))),$$

Cat(·) represents concatenate, and the obtained weight coefficients are multiplied with the original features at the element level, then they are superimposed on the original features to obtain the feature $F_{1}$.

The feature F is more focused on the core information in the channel attention module. We use Avgpool function to obtain the prominent representation of the features and use function to transform the four-dimensional features into two dimensions. The information is extracted by two consecutive linear layers with different activation functions, which are later expanded to the same dimension as feature F. We obtain the weight coefficient $\gamma_{2}$.

(8) $$\gamma_{2}=expand(fc_{2}(fc_{1}(Avg(F)))),$$

expand(·) represents the expand operation on the features, and the obtained weight coefficients are processed in the same way as in the spatial attention module to obtain the feature $F_{2}$.

(9) $$F_{final}=Conv(Cat(F_{1},F_{2}))=Conv(Cat(\gamma_{1}\times F+F,\gamma_{2}\times F+F)),$$

The features $F_{1}$ and $F_{2}$ obtained from the two sub-modules are concatenated, then send to the attention decoder to process the features.

The attention decoder makes the edge of the foreground feature smoother and accelerates the convergence of the model to obtain the final feature $F_{final}$. Then, send it to the final decoder to output the prediction.

### 3.6. Our Network

The decoder of the system decodes the features obtained from the self-enhanced module and the mixed attention module to obtain the prediction output logit_mask. logit_mask is calculated with the target image’s mask target to obtain the loss of the training process, then use it for backpropagation to update the model parameters. logit_mask pass through the argmax function to obtain the final prediction map $P_{red}$.

(10) $$P_{red}=argmax(logit\_mask)$$

The core of few-shot semantic segmentation task is to find similar relations between the query sets and the support sets, so we propose our network to enhance and fuse the representation of similar relations between different modalities. First is the self-enhance of single-modal features, using a series of operations to strengthen the weak connections between similar features, it also amplifies the gap between the foreground feature and the background feature. After that, send it into the mixed attention module to fuse the information between different modalities. It uses coefficient matrices with a different emphasis to strengthen the features. It also extracts the complementary information between different modalities to further enhance the feature. The features are improved gradually until the final feature representation is obtained. In Section 5, the results of experiments further prove our point of view.

## 4. Dataset

For few-shot semantic segmentation tasks, high-quality data are especially important, because the number of samples is too low. Currently, there is a gap in three-modal semantic segmentation datasets both at home and abroad.

To do multi-modal few-shot semantic segmentation, we constructed a multi-modal semantic segmentation dataset VDT-2048-5^i^ and conducted subsequent experiments based on it. The final dataset contains 20 categories, 1037 sets of three-modal images, and their corresponding mask in total. The image sizes are all set to 480 × 640. The dataset and code are available at: https://github.com/VDT-2048/SEMA (accessed on 17 July 2023).

### 4.1. Composition of the Hardware

For RGB-D data acquisition, we use Microsoft’s Kinect V2 camera. Kinect V2 camera can collect the projected infrared reflection and use the Time of Flight (TOF) method to obtain depth information. During the shooting process, RGB images and highly aligned NIR images, and depth images are produced, respectively, which is an important basis for subsequent registration work. Depth, RGB, and NIR images are acquired by installing ROS under Linux and calling iai_kinect2 and libfrekinect packages under ROS.

For the acquisition of thermal infrared images, we use a FLIR thermal imaging camera. This is because the FLIR A655 thermal imaging camera has considerable advantages in temperature measurement accuracy, stability, image quality, and function settings. In addition, the image acquisition method of this camera is relatively simple and can be used by installing the drivers and software from the official website of the FILR camera.

During the processing of V-D-T data, the depth data are more difficult to handle because it is difficult to display the corner points of the checkerboard under normal circumstances. This makes it impossible to use a uniform process for registration directly with V-D-T, which greatly increases the difficulty of the registration process. In this paper, we find that for the Kinect V2 camera’s image generation mode, the NIR images and depth images generated by it can be considered to be fully calibrated, so that the V-D-T registration process can be transformed into a Visible-NIR-TIR registration process, further simplifying the task by such a transformation. When the two cameras are calibrated and registered, the two cameras and the relative position relationship with the collected object are fixed.

**Figure 3:**
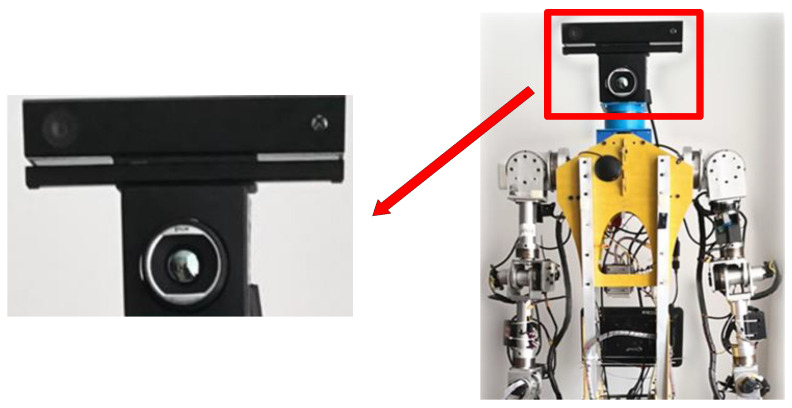
Camera assembly drawing and robot platform.

The two cameras were mounted and kept fixed in the experimental position as shown in Figure 3, including the relative position between the cameras (fixed by the camera stand), the position of the cameras to the robot (fixed by the robot head), and the distance between the cameras and the shooting target (which was indicated by ensuring the position of the robot and the shooting platform), where the shooting distance was set to 2 m by repeated experiments.

### 4.2. Construction of the Dataset

After the registration parameters are obtained, the image data of the three modes of the target are collected without changing the overall position of the hardware. The process flow of this project is as follows:

First, place the hardware in an indoor scene. The object is photographed by category. Then, the multi-modal image is intercepted using the screenshot frame position obtained during the preprocessing process. It does not need to be recalculated because the shooting position and the distance from the lens to the target object remain unchanged. Finally, the thermal infrared and visible images are reconstructed using the previously calculated alignment parameters. Since the relative camera positions remain unchanged, the processing yields highly aligned images.

Specifically, we set up different lighting and different backgrounds for shooting various objects. Three levels of normal light, low light, and dark light are used to imitate the working scenes of the robot in different lighting environments, and different backgrounds are set or blocked to simulate the cluttered working scenes in domestic environments.

In this paper, a total of 20 categories of common items are photographed under different illumination and backgrounds. According to the way the task of few-shot semantic segmentation, to facilitate cross-validation of the algorithm performance, the images are divided the 20 different categories into 4 different folds (sets of subclasses) without repetition as shown in Table 1.

**Figure 4:**
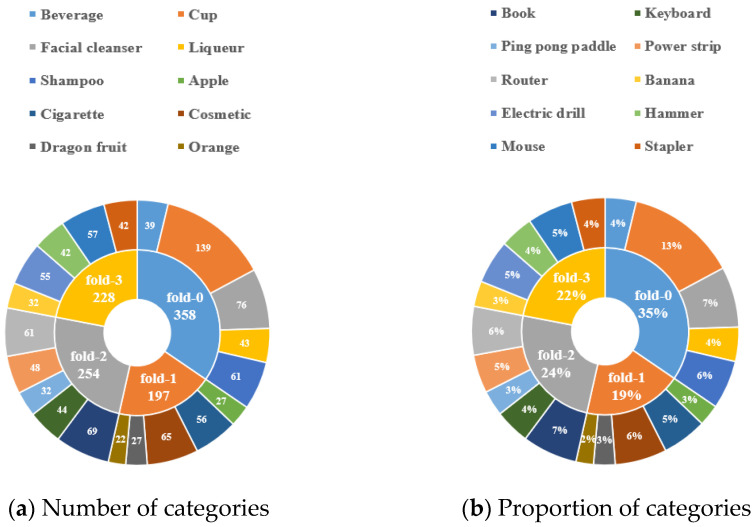
Dataset number and proportion information.

**Figure 5:**
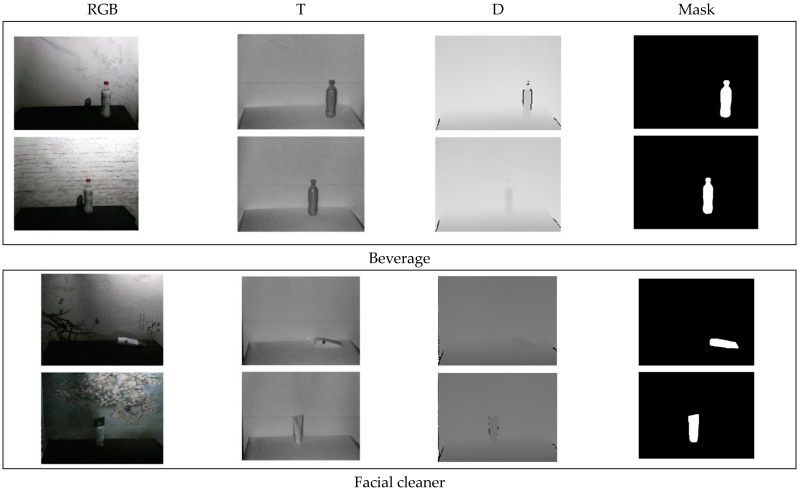
Annotated sample image of dataset.

The number of each category and the corresponding percentages are shown in Figure 4. The annotation aspect of the dataset is similar to the semantic segmentation dataset, all pixels of each image need to be annotated. For the few-shot task, it is considered that only one class of foreground exists for each image, so the final mask image obtained is a binary image with the foreground region being 1 and the background region being 0, as shown in Figure 5. Finally, it should be noted that since the dataset is constructed with insufficient illumination and complex background temperature, so the NIR images are used for image annotation.

## 5. Experiment

### 5.1. Setup Details

We adopted ResNet-50 [60], which pre-trained on ImageNet [62], as the backbone network to extract features. During training phase, we freeze the parameters of the backbone network to reduce the number of parameters and to avoid category bias in the knowledge learned by the model. The network uses the cross-entropy between the prediction image and the ground truth image to generate the loss function, which is used to back propagate to update parameters. The network is implemented in PyTorch [63] and optimized using Adam [64] with learning rate of 1 × 10^−3^. Under the Ubuntu system, we used Nvidia RTX3060TI(8G) for training, and unified the input image size into 400 × 400.

### 5.2. Evaluation Metrics

We use mean intersection over union (mIoU) as our main evaluation metrics, which represents the average *IoU* value of all categories in each fold. It can well reflect the segmentation capability of the model.

The relationship between the ground truth image and the predicted image of can be divided into four types: True Positive (*TP*), False Negative (*FN*), False Positive (*FP*), and True Negative (*TN*), in which True and False represent whether the prediction is correct or not, Positive and Negative represent the foreground and background of the object, respectively.

So, TP represents the intersection of real value and predicted value, *FN* + *FP* + *TP* part is the union of real value and predicted value, and *IoU* is the ratio of intersection and union, which can be expressed as follows:

(11) $$IoU=\frac{TP}{FN+FP+TP},$$

The specific calculation formula of mIoU can be determined as in the following Equation (12), where K presents the number of classes in a fold.

(12) $$mIoU=\frac{1}{K}\sum_{i=1}^{K}\frac{TP}{FN+FP+TP},$$

We also use foreground-background *IoU* (FB-IoU) as an auxiliary metric, which calculate the mIoU of the foreground-background for all categories.

### 5.3. Results and Analysis

We compare our proposed method with relevant methods in recent years [11,37,55,56,57,58,59] in the proposed dataset, VDT-2048-5^i^. Table 2 summarizes the results of the 1-shot and 5-shot comparative experiment on VDT-2048-5^i^. Compared with other methods using ResNet-50, it is clear that our model set a new state-of-the-arts result. Compared with the results of 1 shot and 5 shot in [59], our model increases the mIoU by 3.8% and 3.3%, respectively, which proves the effectiveness of the model.

In order to ensure the fairness of the comparative experiment, all experiments use the same parameter settings on VDT-2048-5^i^ and all experiments are performed with the ResNet-50 backbone.

To test the three-modal effect of the single-modal few-shot semantic segmentation method, we performed the original method on the visible image, the depth image, and the thermal infrared image, respectively. After that, we superimpose the results of the three modalities and feed them into the system’s decoder for prediction, as mentioned in [29].

### 5.4. Ablation Study

In this section, we recombine all the proposed modules with the baseline to verify their effectiveness. All ablation experiments are performed on the VDT dataset with ResNet-50 as the backbone network in 1-shot way. We also discuss the influence of three-modal data.

#### 5.4.1. Ablation Study on Three-Modal Data

To demonstrate the effectiveness of three-modal data, we test different combinations of three modalities six cases in total, three sets of single modality experiments, two sets of two modalities experiments, and one set of three modalities experiments.

To verify the necessity and superiority of the three-modal method, we use the number of parameters to measure the model size and the frames per second (FPS) to measure the running speed of the model. We implement experiments in 1-shot way and report the mean intersection over union over the four folds.

As shown in Table 3, we used several metrics to test the model when using different modalities data as input. We can see that the best result is obtained using RGB images when using single-modal data as input.

The performance of the model improves significantly when an additional modality is added to the input to form two-modal data. The RGB-D combination works slightly better than the RGB-T combination.

The best performance of the model is achieved when three-modal data are used, while the model size is not substantially increased, while the operation time still maintains a better result. In this way, it can be proved that the necessity and superiority of introducing three-modal data into few-shot semantic segmentation task.

#### 5.4.2. Ablation Study on the Proposed Module

For our proposed self-enhanced module and mixed attention module, we set several experiments to prove the efficiency of each module. Specially, we divide the mixed attention module into two parts, spatial attention mechanism module, and channel attention mechanism module.

We implement experiments in 1-shot and report the mean intersection over union over the four folds. In Table 4, the baseline is the backbone of our model, SE is the self-enhanced module, SA is spatial attention mechanism module, and CA is the channel attention mechanism module.

It increases 8.0% mIoU by using the SE module over baseline, which mean the processing of foreground–background features in this module makes it easier for the model to complete the segmentation task.

It increases by 4.2% mIoU by using the SA module. It increases by 5.4% mIoU by using the CA module. And it increases by 6.6% mIoU by using both SA and CA modules, indicating that both SA and CA modules have a reinforcing effect on features and that they can effectively complement each other’s information to better enhance features when used together.

It increases 8.6% mIoU by using SE and SA modules, and it increases 8.7% mIoU by using SE and CA modules, indicating that SA and CA modules can be well compatible with the SE module, and enable better representation of features.

In total, our model improves performance by 10.8% over baseline, which can demonstrate the effectiveness of all proposed modules.

### 5.5. Visualization Results

**Figure 6:**
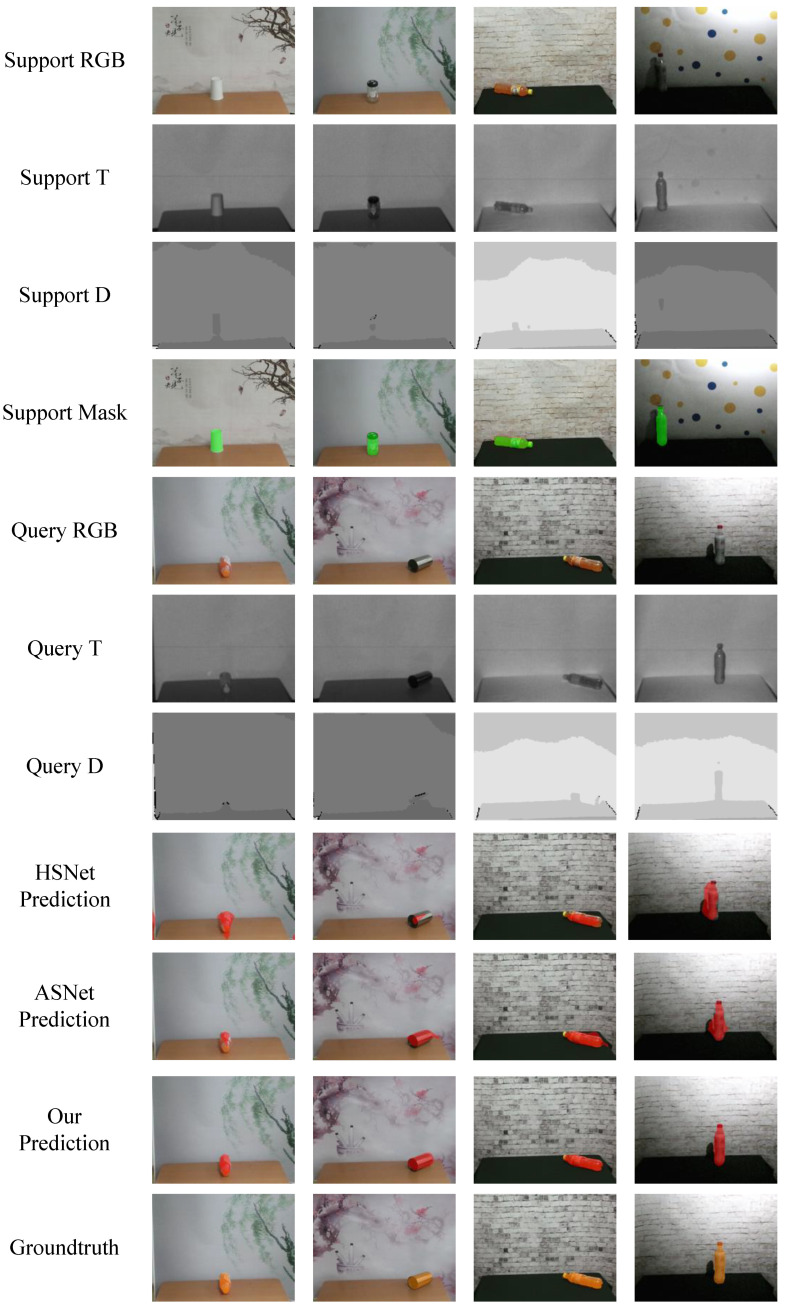
Successful segmentation results in our experiment.

As shown in Figure 6, we measured the effect of our model in a normal illumination scenario. The eighth row are the predicted result of our model which are painted red.

It can be seen from the figure that HSNet and ASNet can only predict part of the target or mistakenly segment the shadow in the background as a part of the target. On the contrary, our model has a good performance in the segmentation results, which shows that our model is valid.

### 5.6. Challenging Scenes

**Figure 7:**
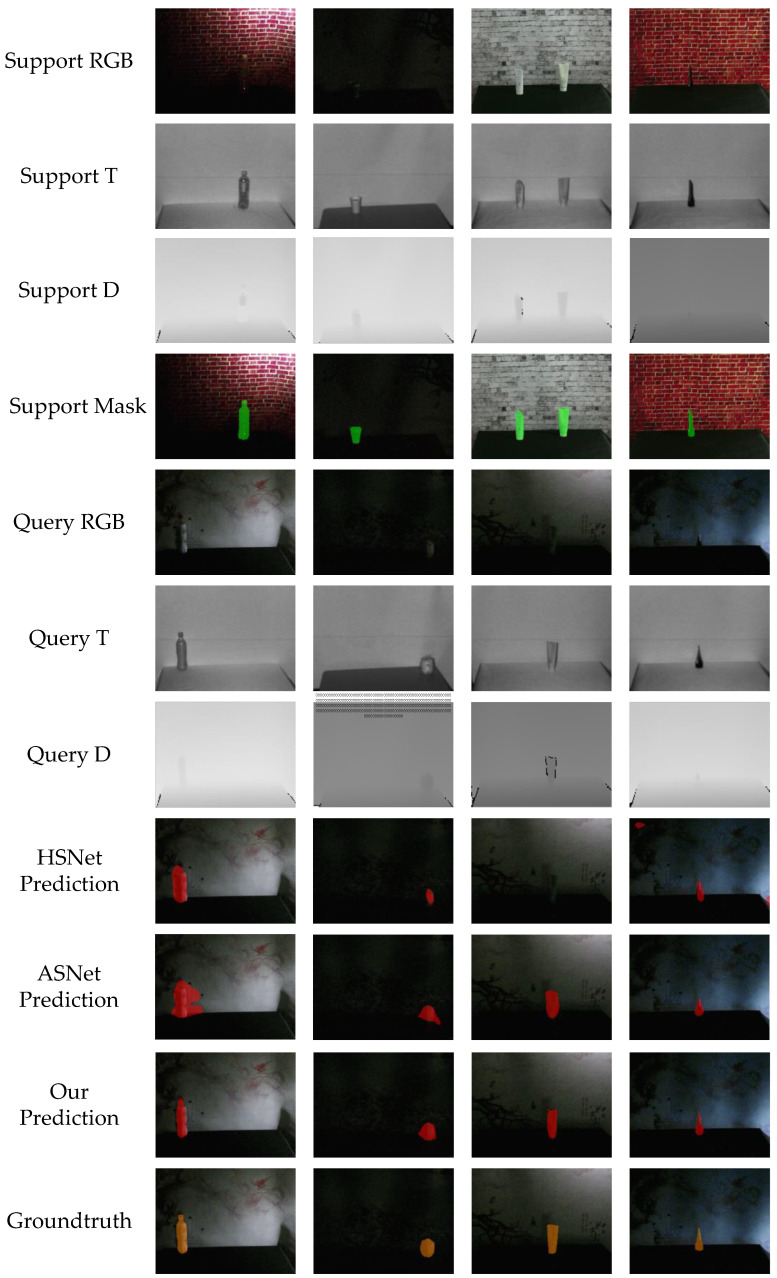
Segmentation result of the challenging scenes in our experiment.

As shown in Figure 7, we conducted a series of experiments in challenging scenes, such as insufficient lighting and tiny objects. The eighth line is the prediction result of our model. Our model can still capture the most discriminating part of the object, even if the supporting image does not provide enough information. This strongly proves the effectiveness of our model. HSNet and ASNet do not perform as well as our model under the condition of insufficient illumination, and they cannot distinguish the boundary between the target and the background well, resulting in a lot of false positives. But, at the same time, we find that the proposed framework is still insufficient in describing pixel-level subtle information such as boundaries or edges, especially in extremely dark small objects. This also provides a direction for our next optimization work.

## 6. Conclusions

In this paper, we introduce three-modal (V-D-T) data into a few-shot semantic segmentation task to improve the performance of the method. As far as we know, our method, SEMANet, is the first and only one. Our SEMANet effectively exploits the complementary information between the three modalities to guide the segmentation, and we achieve excellent performance even with the limited samples. In addition to that, a novel three-modal few-shot dataset is proposed in this paper, VDT-2048-5^i^. Compared with the most advanced methods before, our model improves mIoU by 3.8% and 3.3% in 1-shot and 5-shot settings, respectively, which achieves state-of-the-art performance. The results shows that its effectiveness in the V-D-T FSS task, but, meanwhile, it still has some shortcomings in segmenting the contour of small target objects under dark illumination. In the future, we will optimize for SEMANet by obtaining more discriminative and robust feature representations, and explore achieving high performance with fewer parameters and computational costs.

## Figures and Tables

**Table 1:** Composition of each fold of the VDT-2048-5^i^ dataset.

Fold	Test Classes				
Fold-0	Beverage	Cup	Facial cleanser	Liqueur	Shampoo
Fold-1	Apple	Cigarette	Cosmetic	Dragon fruit	Orange
Fold-2	Book	Keyboard	Ping pong paddle	Power strip	Router
Fold-3	Banana	Electric drill	Hammer	Mouse	Stapler

**Table 2:** Performance on VDT-2048-5^i^ in mIoU and FB-IoU.

Method	1-Way 1-Shot						1-Way 5-Shot					
	5^0^	5^1^	5^2^	5^3^	mIoU	FB-IoU	5^0^	5^1^	5^2^	5^3^	mIoU	FB-IoU
CANet	55.0	55.9	30.4	49.6	**47.7**	72.6	58.7	59.2	41.0	49.8	**52.2**	74.6
PGNet	54.2	56.4	33.6	45.2	**47.4**	71.8	59.7	59.7	34.4	48.5	**50.5**	73.7
PFENet	46.2	45.3	31.8	29.4	**38.2**	67.7	56.5	56.2	43.7	30.7	**46.8**	72.2
ASGNet	40.2	36.2	29.7	36.0	**35.5**	66.5	55.4	51.3	43.8	40.7	**47.8**	72.3
V-TFSS	55.6	53.5	31.0	29.4	**42.4**	72.6	60.6	57.3	45.2	48.5	**52.9**	75.2
HSNet	46.1	45.9	43.3	39.7	**43.8**	71.1	62.0	59.2	48.6	45.0	**53.7**	75.6
ASNet	62.9	57.4	41.0	41.9	**50.8**	73.8	68.5	64.2	48.9	48.5	**57.5**	76.1
Ours	68.9	58.2	49.8	41.3	**54.6**	75.8	74.9	62.2	58.9	47.2	**60.8**	78.9

**Table 3:** Ablation study on three-modal data.

Method	Data	mIoU	FBIoU	Parameter (M)	Time (FPS)
Single modality	RGB	49.2	73.2	31 M	25 FPS
	Depth	30.5	50.8	31 M	25 FPS
	Thermal	37.6	55.2	31 M	25 FPS
Two modalities	RGB-T	51.2	74.5	42 M	17 FPS
	RGB-D	52.2	75.1	42 M	17 FPS
Three modalities	RGB-D-T	54.6	75.8	46 M	14 FPS

**Table 4:** Ablation study on the proposed module.

Baseline	SE	SA	CA	5^0^	5^1^	5^2^	5^3^	mIoU	FB-IoU
√				46.1	45.9	43.3	39.7	**43.8**	71.1
√	√			61.7	55.9	49.3	40.3	**51.8**	74.3
√		√		54.3	49.7	48.5	39.6	**48.0**	72.3
√			√	57.3	50.7	48.8	39.9	**49.2**	73.3
√		√	√	54.0	56.7	49.0	41.8	**50.4**	73.3
√	√	√		65.5	57.0	50.0	37.7	**52.4**	74.9
√	√		√	64.0	56.3	52.1	37.5	**52.5**	74.9
√	√	√	√	68.9	58.2	49.8	41.3	**54.6**	75.8

## Data Availability

To do multi-modal few-shot semantic segmentation, a multi-modal semantic segmentation dataset VDT-2048-5^i^ has been constructed. The final dataset contains 20 categories, 1037 sets of three-modal images, and their corresponding mask in total. The image sizes are all set to 480 × 640. The dataset is available at: https://github.com/VDT-2048/SEMA.
